# Psychological Problems Experienced by Patients with Bowel Endometriosis Awaiting Surgery

**DOI:** 10.1055/s-0041-1735938

**Published:** 2021-10-20

**Authors:** Helizabet Salomão Abdalla Ayroza Ribeiro, Andresa Maria Felipe de Paiva, Beatriz da Costa Porto Taliberti, Anna Luiza Lobão Gonçalves, Renata Pereira Condes, Paulo Augusto Galvão Ayroza Ribeiro

**Affiliations:** 1 Faculdade de Ciências Médicas da Santa Casa de São Paulo, São Paulo, SP, Brazil

**Keywords:** cross-sectional studies, endometriosis, depressive disorders, anxiety disorders, psychological adaptation, estudo transversal, endometriose, transtorno depressivo, transtorno de ansiedade, adaptacão psicológica

## Abstract

**Objective** To assess the most common psychological disturbances in women with deep endometriosis and bowel involvement who are waiting surgical treatment and to evaluate what forms of coping are used to solve the problem.

**Methods** This was a cross-sectional observational study of 40 women diagnosed with deep endometriosis and intestinal symptoms. They completed two questionnaires: one for anxiety and depression (Hospital Anxiety and Depression Scale [ HADS]) and the Scale of Mode of Confronting Problems (EMEP, in the Portuguese acronym).

**Results** We found that 77.1% of the patients had anxiety and depression, with anxiety being the most prevalent (87.5% of the patients); 90% of the patients used problem-focused and religious introspection as their main modes of confronting problems.

**Conclusion** In the use of the HADS questionary, two psychological aspects were the most present in women with deep endometriosis awaiting surgical treatment: anxiety and depression. The most used forms of coping to solve the problem were problem-coping and religious practices.

## Resumo

**Objetivo** Avaliar os distúrbios psicológicos mais comuns em mulheres com endometriose profunda e acometimento intestinal que aguardam tratamento cirúrgico e avaliar as formas de enfrentamento que são usadas para resolver o problema.

**Métodos** Estudo observacional transversal com 40 mulheres com diagnóstico de endometriose profunda e acometimento intestinal. As pacientes responderam dois questionários: para ansiedade e depressão (Hospital Anxiety and Depression Scale [HADS, na sigla em inglês) e outro para enfrentamento dos problemas (EMEP).

**Resultados** Constatamos que 77% das pacientess apresentaram ansiedade e depressão, sendo a ansiedade a mais prevalente (87,5%); 90% das pacientes usaram a forma religiosa e focada no problema como sua principal estratégia de enfrentamento.

**Conclusão** Os aspectos psicológicos mais encontrados em mulheres com endometriose profunda e intestinal que aguardam tratamento cirúrgico são ansiedade e depressão. As formas mais usadas de enfrentamento para resolver o problema foram práticas religiosas e focada no problema.

## Introduction

Endometriosis is the development and growth of endometrial tissue outside the uterine cavity. 1 The overall incidence of endometriosis affects between 6 and 10% of reproductive-aged women and has been found in premenarchal and postmenopausal women. 2 The average age at diagnosis is ∼ 28 years old. Deep endometriosis as the only form of disease in the absence of other endometriotic lesions was present in only 6.5% of the patients. The 5-mm definition permits the inclusion of slightly deeper typical lesions. It would have been preferable to define deep endometriosis. 3 4 5 Bowel infiltration occurs at an incidence ranging from 5 to 12%. 6 7 8

Exams used to diagnose deep endometriosis include transvaginal ultrasonography with bowel preparation and magnetic resonance imaging (MRI) of the pelvis. The typical delay for the diagnosis of endometriosis is of ∼ 6.7 years. 9 10 During this period, patients often suffer from the anguish of not having a diagnosis or of having their complaints devalued. 11 12 13

The most common symptoms of deep endometriosis are the triad dysmenorrhea, dyspareunia, and bowel symptoms, which are present in 80% of the cases of deep bowel endometriosis. Studies suggest that the intensity of pain associated with deep endometriosis does not factor into the staging of the disease. Pain could be the cause of psychosocial problems, loss of productivity at work and changes in quality of life. 12 13 14 15

It is thought that chronic pelvic pain in endometriosis can cause physical, psychological, and social damages just as any chronic disease because it restricts and modifies the daily social life of patients. 16 17 Among these psychological changes, the most common ones associated with chronic pain are anxiety and depression, reported in 60% of the cases. 12 18

Recent studies found that endometriosis and psychological factors can cause a vicious cycle that tends to worsen symptoms such as pelvic pain and can present as anxiety, distress, depression, and stress. 19 20 21

The impact of endometriosis, associated with the persistent painful symptoms, presents with loss of a healthy and active body, subsequently generating a state of dependence and other limitations. 22 Relationships, in general, are harmed because those close to the patient, including family, often get tired of the many complaints of the patients. 23

Because of the diversity of complaints, a multidisciplinary approach that meets both physical and psychological needs of women with endometriosis is critical. Depression is associated with lost work hours and consequent economic losses, and sometimes can result in women being laid off from their jobs because of their complaints of constant discomfort. 24 25 There have been studies on the quality of life of women with deep bowel endometriosis including psychological and physical problems with comparisons before and after surgical procedures; however, these studies did not evaluate patients regarding how they perceive and cope with the disease while awaiting surgery. Therefore, we surveyed women with deep bowel endometriosis to assess their psychological states and coping mechanisms.

The objectives of the present study are to assess the most common psychological disturbances in women with deep endometriosis and bowel involvement who are waiting surgical treatment and to evaluate what forms of coping are used to solve the problem.

## Methods

The present cross-sectional observational study was approved by the Ethics Committee of the Sisterhood of the Santa Casa de Misericórdia de São Paulo, São Paulo, state of São Paulo, Brazil. The ethics committee approved the study under the number 1939017 ( **Appendix 1** ). All patients had a diagnosis of deep endometriosis with bowel involvement, verified using transvaginal ultrasonography for endometriosis mapping. All patients gave informed written consent.

There are 302 women diagnosed with deep endometriosis with bowel compromise who await surgical treatment for about 5 years; all are part of the ambulatory of the Gynecological Endoscopy and Endometriosis department of the Department of Obstetrics and Gynecology of the Santa casa de Misericórdia de São Paulo. After the sample size calculation was done, we randomly selected 40 women considering the inclusion and exclusion criteria.

The instruments used were two questionnaires: one dealing with anxiety and depression (the Hospital Anxiety and Depression Scale [HADS]) 26 27 and the Scale Mode Confrontation of Problems (EMEP, in the Portuguese acronym), 28 29 both of which were chosen by the departments of Gynecological Endoscopy and Psychology of the Santa Casa de Misericórdia of São Paulo to evaluate psychological symptoms and other relevant factors associated with endometriosis, as well as the coping strategies most used by the patients. Another important consideration that affected the choice of instruments was the fact that both questionnaires could be applied by a researcher who was a physician and not a psychologist.

The HADS has its version in Portuguese validated by Botega et al. 26 It is an instrument used both for diagnostic screening and for measuring the severity of anxiety and depression. It is a self-filling scale with seven items for anxiety and seven for depression. The score can range from 0 to 21. Scores above seven are suggestive of anxiety and depression. 26 27

The EMEP was elaborated by Vitaliano et al. 28 and adapted to the Brazilian population by Gimenes et al. 29 The EMEP measures coping strategies in relation to specific stressors. It is self-applicable and contains 45 items distributed into 4 factors: problem-focused coping, emotion-focused coping, search for religious practices, and search for social support. Responses are given on the Likert scale ranging from 1 to 5 points (1 = I never do it; 5 = I do it always). The higher scores indicate greater use of a given coping strategy. 28 29

The study variables were depression, anxiety, and the modes of confronting problems. Based on the EMEP questionary for the modes of confronting problems for the variables are described: strategy 1 (problem-focused strategy), strategy 2 (focused on emotion), strategy 3 (religious introspection), and strategy 4 (social support). The EMEP questionary also evaluates the sequence of options each individual uses to confront a problem (primary, secondary, tertiary, and the last one). 28 29

The sample size was calculated according to a pilot study realized with 5 initial patients who responded the two questionnaires (the HADS and the EMEP). Using a significance level of 5% and a test power of 80% in the assessment of psychological aspects, the questionnaires should be applied to 34 women. Therefore, we set our sample number to 40.

Data were analyzed using IBM SPSS Statistics for Windows version 19.0 (IBM Corp., Armonk, NY, USA). The chi-squared test was used to analyze the data. The level of significance of the tests was 5%.

We included patients with deep endometriosis and bowel compromise awaiting surgery, regardless if they were receiving hormonal treatment. We excluded asymptomatic patients with deep endometriosis and bowel compromise and those without risk of loss of organ function, as well as patients with psychopathologies already under treatment and those taking antidepressants.

## Results

The results are based on the questionnaires applied during the study; the HADS ( Table 1 ) and the EMEP ( Tables 2 , 3 , and 4 ). Due to statistical reasons (low number of responders in some slots), we grouped strategies 1 with 3 (group 1) because these were the modes mostly used to solve the problems by the patients, and strategies 2 with 4 (group 2) because there were the less used modes by the patients.

**Table 1:** Assessment of the anxiety and depression level in women awaiting surgery for deep bowel endometriosis

		Anxiety				Total		Chi-squared test (p)
		Positive		Negative				
		*n*	%	*n*	%	*n*	%	
Depression	Positive	27	77.1%	1	20.0%	28	70.0%	
	Negative	8	22.9%	4	80.0%	12	30.0%	0.037
Total		35	100.0%	5	100.0%	40	100.0%	

**Table 2:** Association between the primary and the secondary modes of confronting problems used by patients with deep endometriosis with bowel compromise, according to the Strategies Mode of Coping Scale (EMEP)

		Secondary mode of confronting problems				Total		Chi-squared test (p)
		Strategies 1 and 3		Strategies 2 and 4				
		*n*	%	*n*	%	*n*	%	
Primary mode of confronting problems	Strategies 1 and 3	27	90.0%	9	90.0%	36	90.0%	
	Strategies 2 and 4	3	10.0%	1	10.0%	4	10.0%	1.000
Total		30	100.0%	10	100.0%	40	100.0%	

**Table 3:** Association between the primary and the last modes of confronting problems used by patients with deep endometriosis with bowel compromise, according to the Strategies Mode of Coping Scale (EMEP)

		Last mode of confronting problems				Total		Chi-squared test (p)
		Strategies 1 and 3		Strategies 2 and 4				
		*n*	%	*n*	%	*n*	%	
First mode of confronting problems	Strategies1 and 3	1	33.3%	35	94.6%	36	90.0%	
	Strategies 2 and 4	2	66.7%	2	5.4%	4	100%	0.016
Total		3	100,0%	37	100.0%	40	100.0%	

**Table 4:** Association between the secondary and tertiary mode of confronting problems used by patients with deep endometriosis with bowel compromise, according to the Strategies of the Mode of Coping with Problems Scale (EMEP)

		Tertiary mode of confronting problems				Total		Chi-squared test (p)
		Strategies 1 and 3		Strategies 2 and 4				
		*n*	%	*n*	%	*n*	%	
Secondary mode of confronting problems	Strategies1 and 3	2	18.2%	28	96.6%	30	75%	
	Strategies 2 and 4	9	81.8%	1	3.4%	10	25%	< 0.001
Total		11	100.0%	29	100.0%	40	100.0%	

Of the total sample selected for the present study, only 4 (10%) patients had neither depression nor anxiety; the remaining 36 (90%) had one of the clinical conditions or both simultaneously ( Table 1 ).

The EMEP identifies the mode of confronting problems with the disease ( Table 2 ). Correlating the primary mode with the secondary, 90% of the women used as the primary and main way of facing the disease strategies 1 and 3, which are, respectively, strategies focused on the problem and the search for religious practices. Only 10% of the patients used strategy 2, which is a form based on emotion, and strategy 4, which used social support as the main coping strategy. However, the crossing between the primary and secondary modes of confronting problems did not present statistical significance.

Table 3 , which shows the EMEP questionnaire results, displays the comparison between the primary and the last modes of confronting problems, according to the choice of the patient, with statistical significance. We found that 90% of these women used strategy 1 (problem-focused strategy) and strategy 3 (religious introspection) as the main modes of confronting problems. As a last mode of confronting problems, they used strategy 2 (focused on emotion) and strategy 4 (social support). Another 10% used the inverse strategy, they used strategy 2 (emotion) and strategy 4 (social support) as the primary mode of confronting problems ( *p* = 0.016), and as the last mode of confronting problems, strategy 1 (problem-focus) and strategy 3 (religious introspection).

The EMEP questionnaire assessed the relationship between the secondary and tertiary modes of confronting problems ( Table 4 ). The secondary mode of confronting problems are the strategies used by patients when the primary one did not solve the problem, and the tertiary mode are the strategies used when the primary and the secondary modes did not solve the problems. Of these women, 75% used strategies 1 (focused on the problem) and 3 (religious introspection) as the secondary mode of confronting problems, and strategies 2 (focused on emotion) and 4 (social support) as the tertiary mode of confronting problems. A reversal of strategies was observed in the remaining 25% of the patients; they used strategies 2 (emotion-focused) and 4 (social support) as the secondary mode of confronting problems, and strategies 1 (problem-focused) and 3 (religious introspection) as the tertiary mode of confronting problems ( *p* < 0.001).

## Discussion

Several studies have reported on the relationship between endometriosis and depression, as well as anxiety disorders; nevertheless, the findings have been inconsistent. 30 31 In the present study, we identified 40 patients, 27 (67.5%) of which suffered from anxiety and depression, 8 (20%) suffered from anxiety only, 1 (2.5%) suffered from depression only, and 4 (10%) did not suffer from anxiety nor depression.

Studies to date have assessed the quality of life of patients with endometriosis mostly using the SF-36 questionnaire, which is a generic questionnaire used to evaluate patients with chronic diseases and is not specific for endometriosis. In the present study, we used questionnaires that are specifically used for the assessment of psychological symptoms (anxiety and depression), the HADS and the EMEP.

It is important to emphasize that one of the primary symptoms of endometriosis is chronic pelvic pain (CPP), which, as in any chronic disease, can cause physical, psychological, and social consequences because it limits and alters the activities of everyday social life. 13 14 The psychological changes most commonly associated with chronic pain are anxiety and depression, which are reported by 60% of the patients. Considering the high incidence of these symptoms, we assessed the psychological characteristics of women with deep endometriosis and bowel involvement using the HADS questionnaire in 40 patients and observed that 35 (87.5%) reported anxiety and 28 (70%) reported depressive symptoms. These rates are consistent with those reported in the literature.

Moreover, 27 patients exhibited anxiety and depression simultaneously, a relatively high rate, corresponding to 77% of the 35 patients. Only 4 of the 40 patients did not report anxiety and depression. 15 16 The annual prevalence of depression in the general population ranges from 3 to 11%, which is much lower than among patients with endometriosis. 23

The strength and uniqueness of the present study, which makes it the first to address this specific topic, was the identification of the coping strategies used by the patients to live with the disease and manage it. Donatti et al. 12 demonstrated that patients with severe levels of depression were influenced regarding decisions about strategies for coping with daily situations; the most depressed patients used nonadaptive strategies for facing stressful situations, while less depressed patients solved their problems in more effective and focused ways. We did not find studies comparing strategies for coping related to endometriosis.

Concerning the mode of confronting problems, the patients used group 1, represented by strategies 1 and 3, corresponding to strategies based on problem-coping and religious practices, respectively. Group 1 was most frequently used as the primary mode of coping, both among patients diagnosed and those not diagnosed with anxiety and depression. In all groups that were compared, the use of group 1 as the primary and secondary modes of confronting problems mostly used was > 50%. In contrast, the group 2 mode of coping, based on emotions and social support (strategies 2 and 4), comprised the two least-used coping mechanisms. Both patients with and without anxiety and depression used group 2 strategies as their tertiary and last modes of confronting problems.

The results of the present study, demonstrating that the patients used group 1 strategies as the primary and secondary modes of confronting problems, follow the personality traits of endometriosis patients reported in the literature. Koninckx et al. 25 described that patients with endometriosis exhibited some common characteristics, such as self-demand, insecurity, intolerance to frustration, omnipotence, and loss of contact with their emotions.

Our findings suggest that the most used coping mechanisms among endometriosis patients are closely related to their personality characteristics, most of which probably develop from the fear of feeling pain and the feeling of uncertainty regarding their future as a result of the disease. This information is important for the management of the disease and to establish clinical and psychological treatments that are more effective in helping patients.

The limitation of our study is the exclusion of patients with psychopathologies already under treatment and of those taking antidepressants, which may be creating a selection bias. Therefore, other studies including this kind of patients are necessary.

## Conclusion

In conclusion, in the use of the HADS questionary, two psychological aspects were the most present in women with deep endometriosis who were not using antidepressants and were awaiting surgical treatment, are anxiety and depression. The most used modes of confronting problems to solve the problem were problem-coping and religious practices.
